# The association of fasting plasma thiol fractions with body fat compartments, biomarker profile, and adipose tissue gene expression

**DOI:** 10.1007/s00726-022-03229-2

**Published:** 2022-12-21

**Authors:** Amany Elshorbagy, Nasser E. Bastani, Sindre Lee-Ødegård, Bente Øvrebø, Nadia Haj-Yasein, Karianne Svendsen, Cheryl Turner, Helga Refsum, Kathrine J. Vinknes, Thomas Olsen

**Affiliations:** 1 Department of Pharmacology, University of Oxford, Oxford, UK; 2 Department of Physiology, Faculty of Medicine, University of Alexandria, Alexandria, Egypt; 3 Department of Nutrition, Institute of Basic Medical Sciences, Faculty of Medicine, University of Oslo, Blindern, Postboks 1046, Oslo, Norway; 4 The Cancer Registry of Norway, Oslo University Hospital, Oslo, Norway

**Keywords:** Low-molecular-weight thiols, Obesity, Truncal adiposity, Android/gynoid ratio, Sulfur amino acids, Aminothiols

## Abstract

People with high plasma total cysteine (tCys) have higher fat mass and higher concentrations of the atherogenic apolipoprotein B (apoB). The disulfide form, cystine, enhanced human adipogenesis and correlated with total fat mass in a Middle-Eastern cohort. In 35 European adults with overweight (88.6% women) and with dual-X-ray absorptiometry measurements of regional fat, we investigated how cystine compared to other free disulfides in their association with total regional adiposity, plasma lipid and glucose biomarkers, and adipose tissue lipid enzyme mRNA (*n* = 19). Most total plasma homocysteine (tHcy) (78%) was protein-bound; 63% of total glutathione (tGSH) was reduced. tCys was 49% protein-bound, 30% mixed-disulfide, 15% cystine, and 6% reduced. Controlling for age and lean mass, cystine and total free cysteine were the fractions most strongly associated with android and total fat: 1% higher cystine predicted 1.97% higher android fat mass (95% CI 0.64, 3.31) and 1.25% (0.65, 2.98) higher total fat mass (both *p* = 0.005). A positive association between tCys and apoB (*β*: 0.64%; 95% CI 0.17, 1.12%, *p* = 0.009) was apparently driven by free cysteine and cystine; cystine was also inversely associated with the HDL-associated apolipoprotein A1 (*β*: −0.57%; 95% CI −0.96, −0.17%, *p* = 0.007). No independent positive associations with adiposity were noted for tGSH or tHcy fractions. Plasma cystine correlated with CPT1a mRNA (Spearman’s *r* = 0.68, *p* = 0.001). In conclusion, plasma cystine—but not homocysteine or glutathione disulfides—is associated with android adiposity and an atherogenic plasma apolipoprotein profile. The role of cystine in human adiposity and cardiometabolic risk deserves investigation. ClinicalTrials.gov identifiers: NCT02647970 and NCT03629392.

**Supplementary Information:**

The online version contains supplementary material available at 10.1007/s00726-022-03229-2.

## Introduction

In the general population, the highest versus lowest plasma total concentrations of the sulfur amino acid cysteine (tCys) are associated with up to 10 kg higher body total fat mass (Elshorbagy et al. 2008), an association that is independent of various confounders and was replicated in multiple populations of varying age and ethnicity (Elshorbagy et al. 2012b, d, 2013a). Evidence from transgenic and dietary animal models suggests that increased cysteine availability is potentially obesogenic (Hasek et al. 2013; Elshorbagy 2014; Niewiadomski et al. 2016; Wanders et al. 2018). Collectively, these findings have lately led to human dietary interventions designed to reduce dietary sulfur amino acid intake with the aim of improving body adiposity and metabolic health (Olsen et al. 2018, 2020a; Stolt et al. 2021).

Plasma tCys includes the sum of free oxidized, free reduced, and protein-bound forms. An oxidized cysteine redox state is associated with ageing, BMI, and cardiovascular disease (CVD) (Oliveira and Laurindo 2018). The epidemiologic association of tCys with adiposity was recently followed up by dissection of which component(s) of the plasma cysteine pool correlates with body fat mass. In 35 healthy adults, tCys in fasting acid-precipitated plasma was on average composed of 62% protein-bound cysteine (bCys), 2% free reduced cysteine, 16% free homogeneous disulfide (cystine), and 20% mixed cysteine disulfides (e.g., cysteine-homocysteine) (Elkafrawy et al. 2021). It was the latter two free oxidized forms that correlated most strongly with fat mass, whereas the major fraction, bCys, did not. Further, ascending physiologic concentrations of cystine enhanced adipogenic differentiation and lipid accumulation in primary culture of human adipose-derived mesenchymal stem cells (Elkafrawy et al. 2021), with implications for fat mass expansion in humans.

These findings suggest that it is not the total body cysteine pool, but rather the free disulfide fraction that is associated with obesity, and with induction of *PPARG* and its target genes during adipogenesis (Elkafrawy et al. 2021). These data warrant replication, and raise several questions. First, it is not known whether other free disulfides in plasma [e.g., homocystine and glutathione (GSH) disulfide (GSSG)] are similarly associated with obesity. Total homocysteine (tHcy) has been thoroughly investigated in relation to BMI, and was found in a systematic review to be modestly higher in individuals with obesity versus normal weight subjects in only 3 out of 8 studies (Wiebe et al. 2018). However, to our knowledge, the association of the different homocysteine and GSH species in plasma has not been investigated. Second, the role of sulfur in adipocytes in rodent models warrants the investigation of circulating tCys, its fractions, and related thiols with human adipose tissue gene expression. Importantly, restricting dietary cysteine (and methionine) availability in rodents alters lipogenic and lipolytic gene expression in adipose tissues, including increased expression of *Acaca, Fasn*, *Scd1*, and *Cpt1a* (Perrone et al. 2010; Hasek et al. 2013) with simultaneous downregulation in liver (Hasek et al. 2013).

A more oxidizing environment is present in both visceral and subcutaneous fat from obese compared to lean individuals (Akl et al. 2017), but body fat depots vary in their association with cardiometabolic risk. Whereas upper body fat/android fat is associated with insulin resistance and CVD risk markers in both sexes (Okosun et al. 2015; Vasan et al. 2018), gynoid fat, and in particular leg fat have been found to be either protective (Vasan et al. 2018), or less strongly associated with metabolic risk as android fat (Okosun et al. 2015). Plasma tCys was associated with insulin resistance in children and adults (Elshorbagy et al. 2012d, 2018) and an adverse lipoprotein profile in adults (Elshorbagy et al. 2012b). In line with previously reported associations with overall obesity, we hypothesize that the cystine fraction of tCys is more strongly associated with android than with gynoid fat mass, markers of impaired glucose metabolism, and an adverse lipid profile.

The present study aims to replicate and extend our recent findings of the association between cystine and total fat mass (Elkafrawy et al. 2021) by exploring, a—the association of plasma tCys fractions with distinct body fat depots that vary in their association with cardiometabolic risk; and b—if other plasma-free disulfide fractions (of homocysteine and/or GSH) are associated with fat mass in healthy adults. For the thiol fractions consistently associated with body fat depots, we explored associations with plasma markers of lipid and glucose metabolism and adipose tissue mRNA expression of genes involved in lipid metabolism in vivo.

## Materials and methods

### Participants

This cross-sectional study utilizes pooled baseline data from healthy free-living individuals with normal weight (*n* = 15) overweight or obesity (*n* = 20) (31 women and 4 men) who were recruited between 2016 and 2018 for participation in two dietary interventions trials with sulfur amino acid restriction (Olsen et al. 2018, 2020a) (Study 1 and Study 2, respectively). All study procedures were similar for both studies and details can be found in the referenced publications (Olsen et al. 2018, 2020a). A brief overview follows.

Inclusion and exclusion criteria were similar for both studies with some exceptions. In Study 1, 15 normal weight men and women aged 20–40 years were recruited, whereas in Study 2, 20 women with overweight and obesity aged 20–40 years were recruited. Exclusion criteria were nearly identical in both studies and included presence of chronic disease or drug use, smoking, veganism, pregnancy or lactation, and intensive physical activity > 3 times per week. An additional exclusion criterion in (Olsen et al. 2018) was high intake of fatty fish or cod liver oil. Because of the similarities in inclusion/exclusion criteria and to maximize variation in body fat, these data were pooled for the current analysis. All participants gave written informed consent. The study was conducted according to the guidelines in the Declaration of Helsinki, and the Regional Ethics Committee for Medical Research in South East Norway both studies. The original studies were registered with ClinicalTrials.gov, with identifiers NCT02647970 and NCT03629392.

### Assessment of body composition

Fasting body composition (lean mass, total fat mass, android fat mass, and gynoid fat mass) measurements were performed with dual-energy X-ray absorptiometry (DXA) using Lunar iDXA (GE Healthcare Lunar, Buckinghamshire, UK) and the software enCORE version 16 including the application CoreScan. System calibration was performed daily and subjects were measured wearing light clothing.

### Anthropometric measurements

Weight and height were measured using a calibrated scale with a built-in stadiometer (Seca 285, Birmingham, UK). Waist circumference was measured at the midpoint between the lower margin of the last palpable rib and the top of the iliac crest. Hip circumference was measured with light clothing and at the widest portion of the buttocks. Each measure was repeated three times and the average used for analysis.

### Blood sampling

Fasting venous blood samples were collected into 2 EDTA-lined vacuum tubes. To trap the thiols, one tube was pre-treated with 150 mmol/L N-ethylmaleimide at 10% of the tube bringing final concentration of N-ethylmaleimide to 15 mmol/L. Blood was centrifuged immediately for 5 min at 4 °C, followed by precipitation with 5-sulfosalicyclic acid 10%, with the resultant supernatant aliquoted and stored at −80 °C until analysis.

### Amino acid assays

Determination of plasma concentrations of tCys, tHcy, tGSH and their respective fractions have been reported previously (Antoniades et al. 2006; Olsen et al. 2018, 2020a). Briefly, all analytes were measured using liquid chromatography–tandem mass spectrometry. Coefficients of variation for total aminothiols were 3.4–6.7%. For the total unbound fraction representing the sum of free reduced and disulfide concentrations, the coefficient of variation ranged from 4 to 6%. The bound fraction was calculated by subtracting the unbound concentration from the total plasma concentration.

### Plasma clinical biochemistry

Measurement of total cholesterol, apolipoprotein-A1 (apoA1), apolipoprotein B (apoB), glucose, and insulin were measured at Department of Medical Biochemistry (Oslo University Hospital Rikshospitalet, Oslo, Norway) by colorimetric and/or enzymatic methods (Cobas c702 analyzer, Roche Diagnostics International Ltd, Rotkreuz, Switzerland). HOMA-IR was calculated as fasting glucose (mmol/l) x fasting insulin (pmol/l)/135.

### Subcutaneous adipose tissue biopsies and quantitative real-time PCR

We explored the associations of thiols with steady-state expression of genes involved in lipid metabolism in white adipose tissue. Information on gene expression was available from the 19 participants in Study 2 and included carnitine palmitoyl transferase 1 (*CPT1A*); sterol regulatory element binding protein 1 (*SREBP*); stearoyl CoA-desaturase 1 (*SCD1*); acetyl CoA-carboxylase (*ACACA*); diacylglycerol acyl transferase 1 (*DGAT1*); fatty acid synthase (*FASN*); peroxisome proliferator-activated receptor γ (*PPARG*) as well as leptin (*LEP*): These genes were selected because they or their circulating markers (i.e., leptin) have been shown to respond to dietary manipulation of cysteine in animals (Perrone et al. 2010; Hasek et al. 2013) and administration of cysteine in in vitro studies (Haj-Yasein et al. 2017; Elkafrawy et al. 2021). Methods for white adipose tissue biopsies and quantitative real-time PCR have been described previously (Olsen et al. 2020a). In brief, subcutaneous white adipose tissue was obtained from the periumbilical region after anaesthesia with a local anaesthetic (5 mL Xylocain 10 mg/mL AstraZeneca, Södertälje, Sweden). After the procedure, biopsies were dissected and snap frozen in liquid nitrogen. Samples were stored at –80 °C until analysis. Prior to analysis, white adipose tissue RNA was isolated using TRIzol (Thermo Fisher Scientific, Waltham, MA, US) and Rneasy lipid Tissue mini kit (Quiagen, Hilden, Germany) according to the manufactured protocol. RNA quantity and quality were checked using a Nanodrop ND-1000 Spectrophotometer (Thermo Fisher Scientific, Waltham, MA, USA). After isolation, 250 ng RNA was reversely transcribed to cDNA using the cDNA Reverse Transcription kit (Applied Biosystems, Waltham, MA, USA). Quantitative real-time PCR was performed with either 2.5 μL diluted cDNA (12.5 ng RNA), a 10 μL Kapa SYBR FAST qPCR Master Mix Universal (KapaBiosystems, Wilmington, MA, US), or 9 μL diluted cDNA (25 ng RNA) and 1 μL predeveloped TaqMan Gene Expression Assays were mixed in 10 μL TaqMan Gene Expression Master Mix (Thermo Fisher Scientific, Waltham, MA, US), on a Bio-Rad CFX96 Touch™ Real-Time PCR Detection System (Bio-Rad Laboratories, Hercules, CA, US). Primer sequences can be found in Supplementary file 1. mRNA levels were normalized to *TBP* and quantified using the ΔΔCt method.

### Statistical analysis

All continuous covariates were log-transformed before analysis. Descriptive statistics are presented as geometric means (gM) and the lower and upper limits of the geometric standard deviation (gSD) as determined by gM/gSD and gM × gSD, respectively. Regression models with body fat compartment as the outcome and total aminothiols and their fractions as the predictor were constructed with age and total lean mass as additional covariates as they are considered important confounders of the association between amino acids and body fat compartments. Regression models with metabolic biomarkers as the outcome were constructed with age as an additional covariate. Adding sex to the models did not alter the estimates and was omitted from the models to avoid overadjustment bias. A sensitivity analysis was conducted where we excluded males (*n* = 4) in the models with body fat compartments as outcome. Spearman’s rank correlation analyses were performed for aminothiols and their fractions with mRNA transcripts in white adipose tissue. We applied the Benjamini–Hochberg multiple tests under an FDR of 0.05 ($Q$). The *p* values from 90 tests on thiols and body composition were ranked ($i$), and a *q* value was calculated for each of the 90 tests ($m$) using the formula $(i/m)Q$. From these calculations, the highest *q* value under the FDR of 0.05 was *q* = 0.048. The original *p* value corresponding to this particular *q* value was 0.011, and tests with *p* < 0.011 were therefore considered significant throughout. All statistical analyses were performed using R version 4.1.0 (R for Statistical Computing, Vienna, Austria).

## Results

### Baseline characteristics

Baseline characteristics, select biomarkers, and plasma thiols with fractions are presented in Table 1. Descriptive characteristics by study are presented in the Supplementary file 2. Briefly, data were obtained from 35 healthy, free-living subjects (31 women and 4 men) with a gM (gSD limits) age of 29.1 (23.7, 35.8) years, BMI of 26.2 (22.4, 30.7) kg/m^2^, and total fat mass of 24.3 (16.0, 36.9) kg.

**Table 1:** Baseline characteristics (*n* = 35)^a^

Age, years	29.1 (23.7, 35.8)
Women, *n* (%)	31 (88.6)
Body adiposity	
BMI, kg/m^2^	26.2 (22.4, 30.7)
Waist circumference, cm	82.8 (73.8, 92.9)
Hip circumference, cm	106 (98.5, 114)
Total fat mass, kg	24.3 (16, 36.9)
Android fat mass, kg	1.58 (0.8, 3.12)
Gynoid fat mass, kg	4.92 (3.23, 7.49)
Android/total fat mass ratio	0.07 (0.05, 0.09)
Gynoid/total fat mass ratio	0.20 (0.18, 0.23)
Plasma biomarkers	
Glucose, mmol/L	4.91 (4.37, 5.51)
Insulin, pmol/L	44.8 (28.6, 70.1)
C-peptide, pmol/L	589 (440, 788)
HOMA-IR	1.63 (1.02, 2.61)
Total cholesterol, mmol/L	4.29 (3.68, 5.01)
Apolipoprotein B, g/L	0.74 (0.60, 0.91)
Apolipoprotein A1, g/L	1.47 (1.21, 1.79)
Triglycerides, mmol/L	0.87 (0.58, 1.28)
Plasma thiols	
Total cysteine, μmol/L	271 (235, 312)
Protein-bound cysteine, μmol/L	131 (107, 160)
Free cysteine, μmol/L	138 (114, 166)
Reduced cysteine, μmol/L	14.8 (10.4, 21)
Cystine, μmol/L	40.2 (34.1, 47.4)
Reduced cysteine/cystine	0.37 (0.28, 0.48)
Total homocysteine, μmol/L	8.31 (6.33, 10.9)
Protein-bound homocysteine, μmol/L	6.47 (4.79, 8.74)
Free homocysteine, μmol/L	1.75 (1.17, 2.61)
Reduced homocysteine, μmol/L	0.19 (0.13, 0.29)
Homocystine, μmol/L	0.02 (0.02, 0.03)
Reduced homocysteine/homocystine	10.7 (6.91, 16.6)
Total glutathione, μmol/L	6.68 (5.07, 8.81)
Protein-bound glutathione, μmol/L	1.44 (0.51, 4.07)
Free glutathione, μmol/L	4.68 (3.38, 6.48)
Reduced glutathione, μmol/L	4.11 (2.82, 5.99)
GSSG, μmol/L	0.05 (0.03, 0.08)
Reduced glutathione/GSSG	85.0 (59.3, 122)

*BMI* body mass index, *GSSG* oxidized glutathione

^a^Continuous data were log-transformed and presented as geometric mean (gSD limits)

**Fig. 1:**
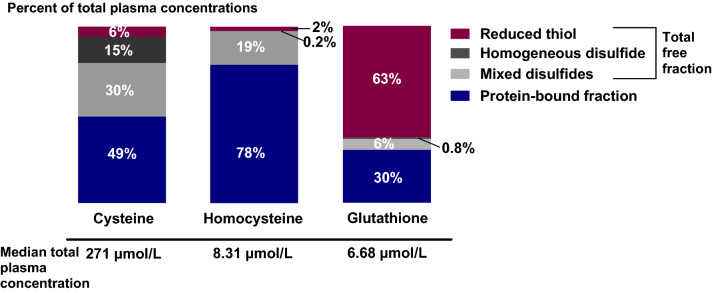
Percentage distribution of plasma total cysteine, total homocysteine, and total glutathione fractions in the study population; *n* = 35. Homogeneous disulfides are, respectively, cystine, homocystine, and glutathione disulfide

Mean plasma concentrations of tCys, tHcy, and tGSH were, respectively, 271, 8.31, and 6.68 μmol/L. The three metabolites varied widely in the relative distribution of the different plasma fractions (Fig. 1). For tCys, 49% was protein-bound, 15% was cystine, 6% was free reduced cysteine, and the remainder was mixed cysteine disulfides. The majority of tHcy was protein-bound (78%), and the majority of tGSH was reduced (63%). For both tHcy and tGSH, homogeneous disulfides constituted less than 1%.

### Correlation among thiols

**Fig. 2:**
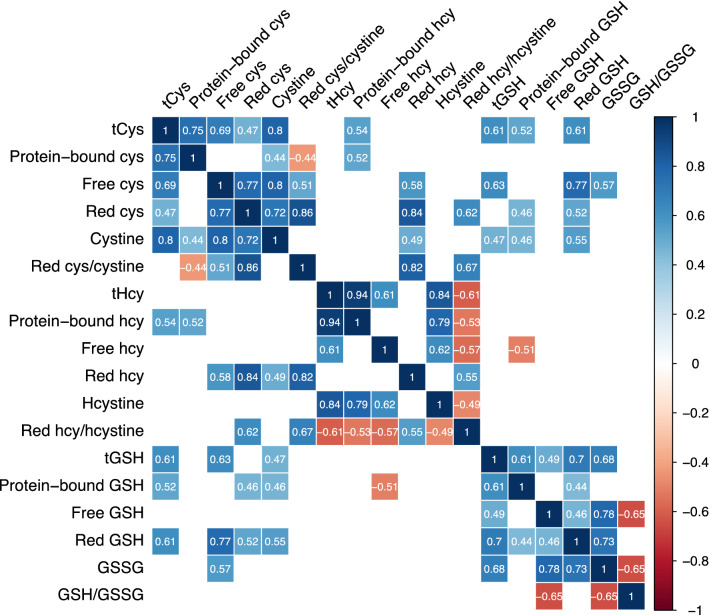
Illustration of correlation coefficients among thiol fractions. Blue tiles denote positive correlations; red tiles denote inverse correlations with a *p* value below 0.011. White (blank) tiles are non-significant. *Cys* cysteine, *GSH* glutathione, *hcy* homocysteine, *Hcystine* homocystine, *Red* reduced, *tCys* total cysteine, *tGSH* total glutathione, *tHcy* total homocysteine

A heatmap showing the Spearman correlation coefficients among the thiols is shown in Fig. 2. Total cysteine correlated with tGSH and the reduced and protein-bound GSH fractions (*r* = 0.52 to 0.61). Reduced cysteine correlated positively with the reduced fractions of homocysteine (*r* = 0.84) and GSH (*r* = 0.77). Reduced homocysteine did not correlate with reduced GSH.

### Thiol fractions and body fat depots

**Fig. 3:**
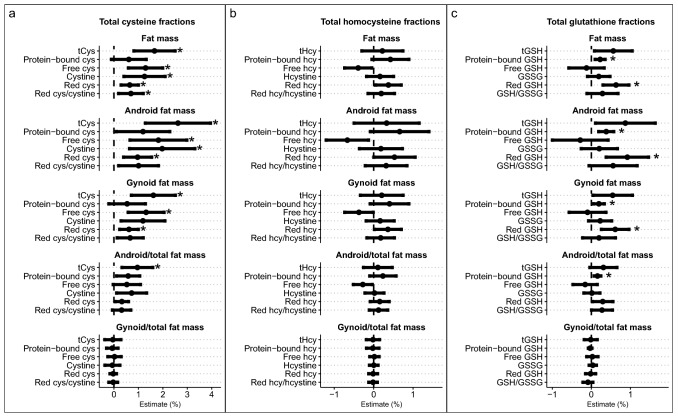
Associations for **a** total cysteine and fractions, **b** total homocysteine and fractions, and **c** total glutathione and fractions with body fat compartments. Estimates were obtained from regression models where log-transformed body fat compartment was the dependent variable and log-transformed thiol was the main independent variable. The models were additionally adjusted for age and lean mass. Estimates indicate % change in body fat compartment per % change in the thiol. Note the differing scales on the X-axes. *cys* cysteine, *GSH* glutathione, *GSSG* oxidized glutathione, *hcy* homocysteine, *Hcystine* homocystine, *tCys* total cysteine, *tHcy* total homocysteine. * indicates *p* < 0.011

Age- and lean mass-adjusted associations for plasma tCys, tHcy, tGSH and their fractions with total fat mass and different body fat compartments as measured by DXA are illustrated in Fig. 3a–c, and estimates and *p* values are available in Online Resource 3.

Plasma tCys concentrations were generally positively correlated with all body fat measures (Fig. 3a; Supplementary file 3). The strongest association was observed for tCys and android fat mass where 1% increase in plasma tCys predicted a 2.62% (95% CI 1.28, 3.95%) increase in android fat mass. With the exception of bCys, all other tCys fractions were consistently and positively associated with total, android, and gynoid fat mass. Of the tCys fractions, the strongest associations were observed for plasma free cysteine (*β*: 1.82%; 95% CI 0.65, 2.98%) and cystine (*β*: 1.97%; 95% CI 0.64, 3.31%) with android fat mass. People with higher plasma tCys had a significantly higher ratio of android fat to total fat mass (*β*: 0.96%; 95% CI 0.33%, 1.59%; *p* < 0.001), but no difference in gynoid/total fat ratio (*p* = 0.78). A similar trend for an association with android/total fat ratio was seen for all cysteine fractions, with cystine having the largest estimate (*β*: 0.72%; 95% CI 0.11%, 1.34%; *p* = 0.023; Fig. 3a; Supplementary file 3). None of the cysteine fractions were associated with gynoid/total fat ratio (*p* ≥ 0.55).

No clear associations were observed for plasma tHcy with body fat compartments.

For tGSH, weak positive associations were observed with total, android, and gynoid fat mass, and android/total fat ratio that were not significant under the adjusted *p* value. These associations appeared to be driven by protein-bound and reduced GSH (Supplementary file 3, Fig. 3c). When we additionally adjusted the models for tCys, all associations were attenuated and no longer significant (Supplementary file 4).

In a sensitivity analysis that included only women (*n* = 31), the results were not appreciably different from the main analysis (data not shown).

### tCys, tGSH fractions, and plasma biomarkers

Because tCys and its free fractions, and the reduced and protein-bound GSH fractions were consistently and positively associated with body fat compartments, we performed additional analyses investigating their associations with markers of an adverse plasma profile including lipoproteins and markers of glucose metabolism. All estimates were adjusted for age, and can be found in Supplementary file 5. For tCys, a positive association was observed with apoB (*β*: 0.64%; 95% CI 0.17, 1.12%, *p* = 0.009). These observations appeared to be driven by free reduced cysteine (*β*: 0.26%; 95% CI 0.07, 0.46%, *p* = 0.010) and cystine (*β*: 0.55%; 95% CI 0.11, 0.99%, *p* = 0.016), although the observation for cystine with apoB was not significant under the adjusted *p* value. Cystine was also inversely associated with apoA1 concentrations (*β*: −0.57%; 95% CI −0.96, −0.17%, *p* = 0.007). No associations were observed for tCys or its fractions with markers of glucose metabolism (glucose, insulin, HOMA-IR, and C-peptide). Reduced GSH was positively association with apolipoprotein B (*β*: 0.24%; 95% CI 0.06, 0.43). No associations were observed for protein-bound cysteine, protein-bound GSH, or for the reduced cysteine/cystine ratio with markers of glucose or lipid metabolism.

### Correlations with adipose tissue gene expression

**Fig. 4:**
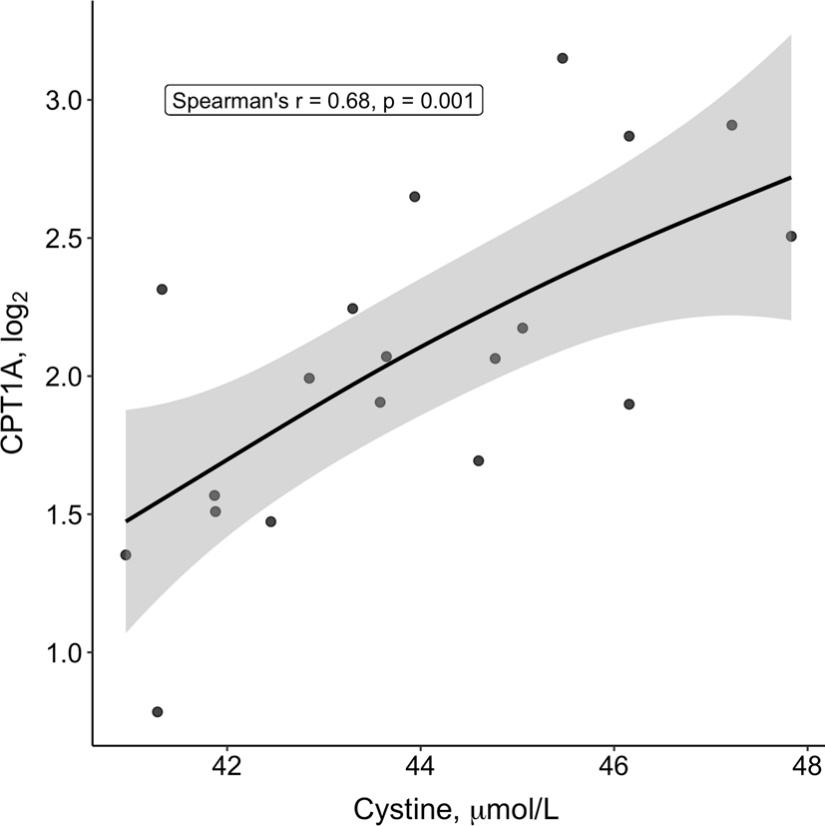
Scatter plot, regression line, and unadjusted Spearman correlation coefficient for the association of plasma cystine with *CPT1A* mRNA transcripts in adipose tissue biopsies. *n* = 19 women

Because tCys and its free fractions as well as protein-bound and reduced GSH were consistently associated with body fat compartments, we performed univariate correlational analyses between the tCys fractions with significant associations and total fat mass, and lipogenic and lipolytic mRNA transcripts in adipose tissue from 19 women with overweight and obesity. Correlation coefficients and *p* values are presented in Supplementary file 6. Plasma concentrations of cystine correlated strongly and positively with *CPT1A* expression (Spearman’s *r* = 0.68, *p* = 0.001; Fig. 4). Inverse correlations were observed for free reduced cysteine with *PPARG* expression (*r* =  −0.53, *p* = 0.021), but this association was not significant under the adjusted *p* value. A similar, but non-significant association was observed for cystine and *PPARG* expression (*r* =  −0.40, *p* = 0.086). No other associations were observed for tCys or other fractions with lipogenic and lipolytic mRNA transcripts. Because expressions of both *CPT1A* and *PPARG* are both associated with body fat mass, we adjusted the correlations for total fat mass. This adjustment did not attenuate the observed correlations (cystine and *CPT1A, r* = 0.67, *p* = 0.002; free reduced cysteine and *PPARG*, *r* =  −0.56, *p* = 0.015 and cystine and *PPARG*, *r* =  −0.46, *p* = 0.055).

## Discussion

The plasma low-molecular-weight thiol pool consists of protein-bound, free oxidized disulfide, and free reduced sulfhydryl forms. Plasma tCys correlates positively with fat mass at the population level, an association that was recently shown to be mediated by its free disulfide forms (Elkafrawy et al. 2021). We sought to test the reproducibility of this finding in a cohort of different ethnicity, to investigate whether other free disulfides similarly correlate with fat mass, and to examine the association of the thiol fractions with regional fat distribution. In 35 adults, total unbound cysteine, cystine, and to a lesser extent, reduced cysteine, were the plasma tCys fractions that correlated positively with fat mass, and this association was not shared by Hcy or GSH disulfides. Of the body fat compartments, tCys and its free forms generally correlated more strongly with android than with gynoid fat, and people with higher tCys and cystine had a higher ratio of android fat to total fat mass. Taken together, these findings indicate an association of tCys with an unhealthy body fat distribution, and that the cystine association with adiposity does not reflect a universal association of circulating low-molecular-weight disulfides with obesity.

In a relatively large study (*n* = 685), plasma cystine increased across BMI categories, but was unrelated to diet quality (Bettermann et al. 2018). Subsequently, we observed that cystine and mixed cysteine disulfides correlate with total fat mass measured by bioimpedance (Elkafrawy et al. 2021). Here, we confirm these findings using DXA, and show that tCys and its free fractions correlate most strongly with android body fat. This finding in a predominantly female young adult cohort is consistent with previous observations that plasma tCys was independently associated with trunk fat/total fat ratio both in 950 children with overweight (Elshorbagy et al. 2012d) and in 610 older adults (Elshorbagy et al. 2013a). Android obesity is a recognized correlate of cardiometabolic risk markers (Vasan et al. 2018) and an independent predictor of future diabetes (Brahimaj et al. 2019). Plasma tCys was also associated with visceral fat area measured by CT (Elshorbagy et al. 2018), and was found recently to predict incident diabetes in older adults (Elshorbagy et al. 2022). Overall, tCys and its free fractions were elevated in individuals with central adiposity in the present study. This finding should be viewed in the wider context of the existing premise that high plasma cystine is a marker of oxidative stress: studies have linked high cystine and low rGSH to multiple adverse outcomes, including CVD pathogenesis (Go and Jones 2011), renal disease progression (Rodrigues et al. 2012), and mortality (Patel et al. 2016).

In line with its association with android adiposity, tCys correlated positively with the atherogenic apolipoprotein apoB, and inversely with apoA1, the main antiatherogenic protein component of HDL particles (German and Shapiro 2020). A higher ratio of apoB to apoA1 is a recognized risk factor for cardiovascular disease (Walldius and Jungner 2006). The positive and negative associations of tCys with apoB and apoA1, respectively, were previously observed in our study of 850 European adults (Elshorbagy et al. 2012b). The present study extends these findings by showing that these associations appear to be mediated by the free forms of tCys including free reduced cysteine and cystine. In rats, a high-cystine diet increased circulating concentrations of the atherogenic apolipoprotein B (apoB), with a trend for raising apoB protein and mRNA expression in liver (Sérougne et al. 1995). tCys and its fractions, however, were unrelated to glucose metabolism biomarkers. We have noted from different datasets that over the lower end of the population range of insulinemia (median ~ 45 pmol/L: (Elshorbagy et al. 2017) and the present study), tCys did not correlate with insulin resistance, but that it was associated with insulin resistance in populations with higher insulin (mean 69 and 98 pmol/L (Elshorbagy et al. 2012d, 2018). Overall, the association of tCys with glucose biomarkers appears less consistent than with adiposity, and is more evident when impaired glucose metabolism is present.

Given previous findings that cystine influences adipogenic gene expression during adipogenesis in murine (Haj-Yasein et al. 2017) and human (Elkafrawy et al. 2021) cells, we investigated whether, in vivo, the cysteine forms that correlated with fat mass are associated with adipose tissue expression of lipid metabolism genes*.* The panel of genes in the present study overlapped with genes whose induction was influenced by cystine in vitro in two key genes, *PPARG* and *SCD1.* In rodents, changes in cysteine availability due to gene knockouts or dietary modifications have substantial and tissue-specific effects on both *Pparg* (Elshorbagy et al. 2012a) and *Scd1* expression (Elshorbagy et al. 2011, 2013b; Gupta et al. 2013; Elshorbagy 2014), dependent on model and experimental setting. In both the mouse-derived 3T3-L1 cell line (Haj-Yasein et al. 2017), and in human primary preadipocytes, increasing extracellular cystine enhances *PPARG* and *SCD1* expression during adipogenesis, with a six-to-sevenfold difference in their degree of induction in human preadipocytes across cystine concentrations (Elkafrawy et al. 2021). However, in the present study, there was no correlation between plasma cystine and adipose tissue *PPARG* or *SCD1* expression. This suggests that while cystine might influence the degree of induction of *PPARG* and its target genes in response to adipogenic stimuli in differentiating preadipocytes (Elkafrawy et al. 2021), cystine may be unrelated to their steady-state expression in mature human adipocytes.

Individuals with higher plasma cystine in the present cohort had a higher adipose tissue *CPT1A* mRNA level. CPT1a catalyzes the rate-limiting step of converting long-chain fatty acids into acyl-carnitines, which can then cross the mitochondrial membrane for oxidation in the mitochondria. In rodent studies, limiting dietary cysteine availability increased CPT1a mRNA and protein expression in inguinal adipose tissue (Perrone et al. 2010) (in line with the anti-obesity effects of the diet), but not in liver (Yang et al. 2019). In our short-term sulfur amino acid restriction trial in humans, however, no significant effect on adipose tissue *CPT1A* was noted despite a nearly fourfold difference in sulfur amino acid intake between the low- and high-intake groups (Olsen et al. 2020a). Although it is a key enzyme in fatty acid oxidation, *CPT1A* expression in human adipose tissue is positively associated with BMI in cross-sectional analysis (Warfel et al. 2017). We therefore hypothesized that the association of cystine with *CPT1A* expression in the present study may partly be explained by their respective links to adiposity, but adjusting for fat mass did not attenuate the association. It is therefore presently unclear whether the observed association between *plasma* cystine and *CPT1A* expression is physiologically relevant in humans, and this link requires replication and, if confirmed, investigation of possible mechanisms. In addition, *CPT1A* expression may not correlate with CPT1A protein abundance, although dietary cysteine restriction increased both expression and protein levels (Perrone et al. 2010), suggesting a positive correlation between mRNA and protein levels.

No consistent associations were observed for plasma homocysteine or GSH species with fat mass, apart from a positive association of bGSH and rGSH with total fat and android fat. The explanation for this finding is unclear, and conflicts with previous observations that tGSH correlates inversely with percent body fat in adults (Elshorbagy et al. 2022) and children (Elshorbagy et al. 2012d). The finding could be partly related to the current assay method involving addition of N-ethylmalemide into the blood tube, which immediately traps all the thiol species. This not only prevents oxidation of plasma thiols but also reduces the rapid redox changes that occur after blood collection (Mansoor et al. 1992); hence, it is worth verifying this finding in other cohorts where the same method is used. We also postulate that this positive association of rGSH with adiposity may be partly driven by the uniquely strong correlation of GSH species with tCys in the current dataset, since adjusting for tCys weakened the association. In other larger cohorts, we have typically found no association between tCys and tGSH in either adults or children (Elshorbagy et al. 2012d, c). In these cohorts, tGSH showed no or negative associations with adiposity (Elshorbagy et al. 2012d, c), in line with findings from other groups (Bettermann et al. 2018). We note that the GSH/GSSG and reduced cysteine/cystine redox pairs have been reported to be in disequilibrium and differentially affected by various interventions, indicating that the observed attenuation may be brought about by an unknown and unmeasured confounding factor (Jones 2006) or other properties that are unique to this dataset such as the small sample size.

Relative concentrations of plasma thiol fractions vary widely with food intake, in a time- and meal-type-dependent fashion (Park et al. 2010; Olsen et al. 2020b). The propensity for plasma reduced thiols to undergo rapid autooxidation also necessitates acid-precipitation following blood withdrawal to preserve the redox forms of the different fractions and enable accurate quantification. Partly owing to these considerations, plasma thiol fractions are generally absent from high-throughput plasma amino acid assays in large cohorts, many of which are based on stored and often non-fasting samples. The present study utilizes overnight-fasted samples that were immediately acid-precipitated and kept cold during centrifugation. This has ensured that the measured thiol fractions are as representative as possible of their steady-state circulating concentrations, and avoided spurious variability due to prandial state and sample treatment—but comes at the expense of the small cohort size. That regional fat estimates were from gold standard DXA measures further enhances reliability of the findings. Most participants were women, which minimizes residual confounding due to sex on the associations seen, but it should be noted that the tCys association with fat mass is marginally stronger in women than men (Elshorbagy et al. 2008). The relation of cysteine forms to adipose tissue gene expression was only tested in a small subset of 19 women, so the null associations for some genes may result from being underpowered for this exploratory analysis, and further studies are needed.

In summary, the present study shows that the positive association of plasma cystine with total fat mass is linked to android adiposity and an atherogenic plasma lipoprotein profile; associations that are not shared by other free lower molecular weight disulfides in plasma. Studies are needed to investigate the determinants of plasma tCys and the balance between its free and protein-bound fractions in humans, and whether cystine, given its associations with an unhealthy body fat distribution and lipoprotein profile, predicts cardiometabolic outcomes.

## Supplementary Information

Below is the link to the electronic supplementary material.

Supplementary file 1 (DOCX 14 KB)

Supplementary file 2 (DOCX 18 KB)

Supplementary file 3 (DOCX 22 KB)

Supplementary file 4 (DOCX 14 KB)

Supplementary file 5 (DOCX 19 KB)

Supplementary file 6 (DOCX 16 KB)

## Data Availability

Data can be shared upon reasonable request to the corresponding author, and is subject to ethical approvals and privacy regulations.
