# Correction to: The association of fasting plasma thiol fractions with body fat compartments, biomarker profile, and adipose tissue gene expression

**DOI:** 10.1007/s00726-023-03244-x

**Published:** 2023-02-16

**Authors:** Amany Elshorbagy, Nasser E. Bastani, Sindre Lee-Ødegård, Bente Øvrebø, Nadia Haj-Yasein, Karianne Svendsen, Cheryl Turner, Helga Refsum, Kathrine J. Vinknes, Thomas Olsen

**Affiliations:** 1 Department of Pharmacology, University of Oxford, Oxford, UK; 2 Department of Physiology, Faculty of Medicine, University of Alexandria, Alexandria, Egypt; 3 Department of Nutrition, Institute of Basic Medical Sciences, Faculty of Medicine, University of Oslo, Blindern, Postboks 1046, Oslo, Norway; 4 The Cancer Registry of Norway, Oslo University Hospital, Oslo, Norway

**Correction to: Amino Acids** 10.1007/s00726-022-03229-2

In Fig. 3 headers were missing in the original article. The correct Fig. 3 is below. 

The original article has been corrected.

**Fig. 3:**
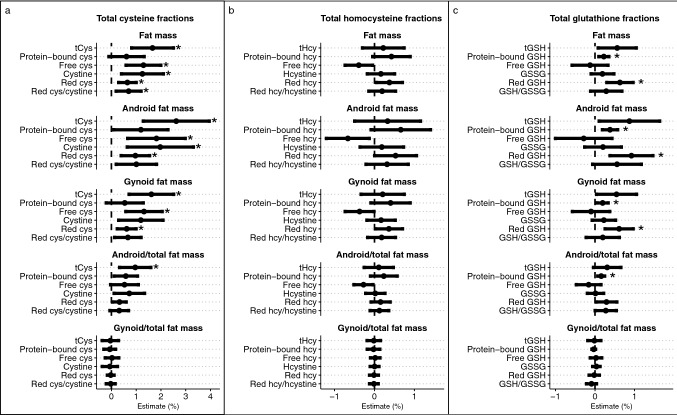
Associations for **a** total cysteine and fractions, **b** total homocysteine and fractions, and **c** total glutathione and fractions with body fat compartments. Estimates were obtained from regression models where log-transformed body fat compartment was the dependent variable and log-transformed thiol was the main independent variable. The models were additionally adjusted for age and lean mass. Estimates indicate % change in body fat compartment per % change in the thiol. Note the differing scales on the X-axes. *cys* cysteine, *GSH* glutathione, *GSSG* oxidized glutathione, *hcy* homocysteine, *Hcystine* homocystine, *tCys* total cysteine, *tHcy* total homocysteine. * indicates *p* < 0.011

