# Supplementary material for: The association of fasting plasma thiol fractions with body fat compartments, biomarker profile, and adipose tissue gene expression
Source: Amino Acids. 2022 Dec 21;55(3):313–23. doi: 10.1007/s00726-022-03229-2 (PMC10038976; doi:10.1007/s00726-022-03229-2)
Supplement: Supplementary file 1 — (DOCX 14 KB) [file 726_2022_3229_MOESM1_ESM.docx]

| **Online Resource 1. Primer sequences of gene expression measured by qPCR** | |
| --- | --- |
| Primer | Sequence |
| *ACACA* | \| ATTGGGGCTTACCTTGTCCG \| \| --- \| \| CGAGGACTTTGTTGAGGGCT \| |
| *CPT1A* | \| ATGTACGCCAAGATCGACCC \| \| --- \| \| GACATGCAGTTGGCCGTTTC \| |
| *DGAT1* | \| AGCCCTTCAAGGACATGGAC \| \| --- \| \| GATGAGGTGATTGGGGACCG \| |
| *FASN* | \| CTTCAAGGAGCAAGGCGTGA \| \| --- \| \| ACTGGTACAACGAGCGGATG \| |
| *LEP* | ACCACCCCCAAATTTT  AGATTAGTAGAGAAGGAGGAAGGA |
| *PPARG* | \| ACAGATCCAGTGGTTGCAGA \| \| --- \| \| TCCACTTTGATTGCACTTTGGT \| |
| *SCD1* | \| CTGCAGGACGATATCTCTAGCTC \| \| --- \| \| TCCAAGTAGAGGGGCATCGT \| |
| *SREBP* | \| CCGCTCCTCCATCAATGACA \| \| --- \| \| GCTGTGTTGCAGAAAGCGAA \| |
| *TBP* | GTGGGGAGCTGTGATGTGAA  ACCAGGAAATAACTCTGGCTCA |
