# Supplementary material for: The association of fasting plasma thiol fractions with body fat compartments, biomarker profile, and adipose tissue gene expression
Source: Amino Acids. 2022 Dec 21;55(3):313–23. doi: 10.1007/s00726-022-03229-2 (PMC10038976; doi:10.1007/s00726-022-03229-2)
Supplement: Supplementary file 2 — (DOCX 18 KB) [file 726_2022_3229_MOESM2_ESM.docx]

**Online resource 2: Baseline characteristics of Study 1 (n = 15) and Study 2 (n = 20)^a^**

|  | Study 1 | Study 2 |
| --- | --- | --- |
| Age, y | 27 (21.7, 33.6) | 30.6 (25.3, 36.9) |
| Women, n (%) | 11 (78.6) | 20 (100) |
| *Body adiposity* |  |  |
| BMI, kg/m^2^ | 22.6 (21.1, 24.2) | 28.9 (25.7, 32.5) |
| Waist circumference, cm | 74.3 (69.2, 79.8) | 88.6 (81.8, 96) |
| Hip circumference, cm | 99.2 (94.5, 104) | 110 (104, 116) |
| Total fat mass, kg | 17 (12.7, 22.7) | 30.8 (22.6, 41.9) |
| Android fat mass, kg | 0.89 (0.55, 1.43) | 2.32 (1.41, 3.82) |
| Gynoid fat mass, kg | 3.55 (2.57, 4.9) | 6.11 (4.39, 8.50) |
| Android/gynoid fat mass ratio | 0.25 (0.18, 0.35) | 0.38 (0.27, 0.54) |
| Android/total fat mass ratio | 0.05 (0.04, 0.07) | 0.08 (0.06, 0.10) |
| Gynoid/total fat mass ratio | 0.21 (0.18, 0.24) | 0.20 (0.18, 0.23) |
| *Plasma biomarkers* |  |  |
| Glucose, mmol/L | 4.71 (4.06, 5.46) | 5.05 (4.66, 5.47) |
| Insulin, pmol/L | 41.3 (25.6, 66.5) | 47.3 (30.7, 72.8) |
| C-peptide, pmol/L | 553 (399, 766) | 615 (472, 801) |
| HOMA-IR | 1.44 (0.86, 2.40) | 1.77 (1.15, 2.73) |
| Total cholesterol, mmol/L | 4.12 (3.52, 4.82) | 4.40 (3.79, 5.11) |
| Apolipoprotein B, g/L | 0.65 (0.56, 0.76) | 0.80 (0.65, 0.99) |
| Apolipoprotein A1, g/L | 1.58 (1.32, 1.89) | 1.41 (1.16, 1.72) |
| Triglycerides, mmol/L | 0.71 (0.52, 0.97) | 0.98 (0.66, 1.47) |
| *Plasma thiols* |  |  |
| Total cysteine, μmol/L | 245 (216, 278) | 294 (270, 321) |
| Protein-bound cysteine, μmol/L | 129 (109, 152) | 135 (109, 168) |
| Free cysteine, μmol/L | 116 (104, 130) | 156 (138, 176) |
| Reduced cysteine, μmol/L | 11.0 (8.65, 14.0) | 18.2 (14.1, 23.5) |
| Cystine, μmol/L | 35.6 (29.6, 42.9) | 44.1 (41.8, 46.5) |
| Reduced cysteine/Cystine | 0.31 (0.25, 0.39) | 0.41 (0.33, 0.52) |
| Total homocysteine, μmol/L | 8.11 (6.09, 10.8) | 8.46 (6.43, 11.1) |
| Protein-bound homocysteine, μmol/L | 5.86 (4.23, 8.12) | 6.98 (5.31, 9.17) |
| Free homocysteine, μmol/L | 2.27 (1.78, 2.89) | 1.44 (0.99, 2.10) |
| Reduced homocysteine, μmol/L | 0.14 (0.10, 0.20) | 0.23 (0.16, 0.34) |
| Homocystine, μmol/L | 0.02 (0.01, 0.03) | 0.02 (0.01, 0.03) |
| Reduced homocysteine/homocystine | 8.94 (5.13, 15.6) | 11.9 (8.63, 16.4) |
| Total glutathione, μmol/L | 5.5 (4.72, 6.41) | 7.75 (5.99, 10.0) |
| Protein-bound glutathione, μmol/L | 0.66 (0.26, 1.65) | 2.48 (1.2, 5.12) |
| Free glutathione, μmol/L | 4.65 (3.67, 5.90) | 4.69 (3.19, 6.89) |
| Reduced glutathione, μmol/L | 11.0 (8.65, 14.0) | 5.36 (4.49, 6.4) |
| GSSG, μmol/L | 0.04 (0.03, 0.05) | 0.06 (0.04, 0.10) |
| Reduced glutathione/GSSG | 74.8 (57, 98.1) | 90.2 (61.1, 133) |

^a^ Continuous data were log-transformed and presented as geometric mean (gSD limits). Abbreviations: BMI, body mass index; GSSG, oxidized glutathione
