# Supplementary material for: The association of fasting plasma thiol fractions with body fat compartments, biomarker profile, and adipose tissue gene expression
Source: Amino Acids. 2022 Dec 21;55(3):313–23. doi: 10.1007/s00726-022-03229-2 (PMC10038976; doi:10.1007/s00726-022-03229-2)
Supplement: Supplementary file 3 — (DOCX 22 KB) [file 726_2022_3229_MOESM3_ESM.docx]

**Online Resource 3: Regression estimates and confidence intervals for the association of total thiol and fractions with body fat compartments^a^**

|  |  | **Total fat mass** | **Android fat mass** | **Android/Total fat mass** | **Gynoid fat mass** | **Gynoid/Total fat mass** | |  |
| --- | --- | --- | --- | --- | --- | --- | --- | --- |
| **Cysteine** | | | | | | | |  |
|  | **Total** | **1.66 (0.81, 2.50)** | **2.62 (1.28, 3.95)** | **0.96 (0.33, 1.59)** | **1.61 (0.71, 2.51)** | -0.05 (-0.38, 0.29) | |  |
|  |  | **p < 0.001** | **p < 0.001** | **p < 0.001** | **p = 0.001** | p = 0.78 | |  |
|  | **Protein-bound** | 0.61 (-0.10, 1.32) | 1.19 (0.09, 2.29) | 0.58 (0.11,1.05) | 0.54 (-0.21, 1.29) | -0.07 (-0.31,0.17) | |  |
|  |  | p = 0.092 | p = 0.034 | p = 0.018 | p = 0.15 | p = 0.55 | |  |
|  | **Free** | **1.29 (0.59, 2.00)** | **1.82 (0.65, 2.98)** | 0.53 (-0.04,1.09) | **1.31 (0.58, 2.05)** | 0.02 (-0.25,0.3) | |  |
|  |  | **p < 0.001** | **p = 0.003** | p = 0.07 | **p = 0.001** | p =0.87 | |  |
|  | **Disulfide (cystine)** | **1.25 (0.40, 2.10)** | **1.97 (0.64, 3.31)** | 0.72 (0.11,1.34) | **1.19 (0.29, 2.08)** | -0.06 (-0.37,0.24) | |  |
|  |  | **p = 0.005** | **p = 0.005** | p =0.023 | **p = 0.011** | p = 0.674 | |  |
|  | **Reduced** | **0.64 (0.29, 1.00)** | **0.97 (0.39, 1.54)** | 0.32 (0.05,0.6) | **0.61 (0.23, 1)** | -0.03 (-0.17,0.11) | |  |
|  |  | **p < 0.001** | **p = 0.002** | p = 0.023 | **p = 0.003** | p = 0.66 | |  |
|  | **Reduced cys/cystine** | **0.69 (0.19, 1.20)** | 1.01 (0.19, 1.82) | 0.31 (-0.07,0.69) | 0.66 (0.13, 1.20) | -0.03 (-0.21,0.15) | |  |
|  |  | **p = 0.009** | p = 0.017 | p = 0.10 | p = 0.017 | p = 0.74 | |  |
| **Homocysteine** | | | | | | |  | |
|  | **Total** | 0.22 (-0.30, 0.74) | 0.33 (-0.50, 1.15) | 0.11 (-0.26,0.47) | 0.21 (-0.34, 0.75) | -0.02 (-0.19,0.15) | |  |
|  |  | p = 0.39 | p = 0.42 | p = 0.55 | p = 0.45 | p = 0.84 | |  |
|  | **Protein-bound** | 0.43 (-0.05, 0.90) | 0.66 (-0.08, 1.40) | 0.23 (-0.1,0.57) | 0.4 (-0.09, 0.89) | -0.02 (-0.18,0.14) | |  |
|  |  | p = 0.075 | p = 0.080 | p = 0.16 | p = 0.11 | p = 0.77 | |  |
|  | **Free** | -0.39 (-0.74, -0.05) | -0.67 (-1.2, -0.13) | -0.27 (-0.51,-0.03) | -0.37 (-0.74, -0.01) | 0.02 (-0.1,0.14) | |  |
|  |  | p = 0.027 | p = 0.017 | p = 0.026 | p = 0.044 | p = 0.75 | |  |
|  | **Disulfide (homocystine)** | 0.16 (-0.18, 0.51) | 0.18 (-0.36, 0.73) | 0.02 (-0.22,0.26) | 0.17 (-0.19, 0.52) | 0.00 (-0.11,0.11) | |  |
|  |  | p = 0.34 | p = 0.50 | p = 0.86 | p = 0.35 | p = 0.96 | |  |
|  | **Reduced** | 0.37 (0.05, 0.70) | 0.53 (0.00, 1.05) | 0.15 (-0.09,0.39) | 0.36 (0.02, 0.70) | -0.01 (-0.13,0.1) | |  |
|  |  | p = 0.026 | p = 0.049 | p =0.20 | p = 0.039 | p = 0.82 | |  |
|  | **Reduced hcy/hcystine** | 0.19 (-0.14, 0.53) | 0.32 (-0.21, 0.85) | 0.12 (-0.11,0.36) | 0.18 (-0.17, 0.53) | -0.01 (-0.12,0.1) | |  |
|  |  | p = 0.25 | p = 0.23 | p = 0.29 | p = 0.31 | p = 0.79 | |  |
| **Glutathione** | | | | | | |  | |
|  | **Total** | 0.56 (0.08, 1.04) | 0.87 (0.10, 1.63) | 0.31 (-0.04,0.66) | 0.54 (0.04, 1.05) | -0.02 (-0.18,0.15) | |  |
|  |  | p = 0.024 | p = 0.027 | p = 0.08 | p = 0.036 | p = 0.84 | |  |
|  | **Protein-bound** | **0.22 (0.1, 0.35)** | **0.38 (0.19, 0.57)** | **0.16 (0.07,0.24)** | **0.19 (0.05, 0.33)** | -0.03 (-0.08,0.02) | |  |
|  |  | **p = 0.001** | **p < 0.001** | **p = 0.001** | **p = 0.010** | p = 0.18 | |  |
|  | **Free** | -0.12 (-0.58, 0.33) | -0.28 (-1.00, 0.43) | -0.16 (-0.47,0.15) | -0.1 (-0.57, 0.37) | 0.03 (-0.12,0.17) | |  |
|  |  | p = 0.58 | p = 0.42 | p = 0.31 | p = 0.67 | p = 0.72 | |  |
|  | **Disulfide (GSSG)** | 0.19 (-0.10, 0.48) | 0.2 (-0.26, 0.67) | 0.01 (-0.19,0.22) | 0.22 (-0.07, 0.52) | 0.04 (-0.06,0.13) | |  |
|  |  | p = 0.19 | p = 0.38 | p = 0.90 | p = 0.13 | p = 0.451 | |  |
|  | **Reduced** | **0.63 (0.30, 0.96)** | **0.92 (0.38, 1.46)** | 0.29 (0.03,0.55) | **0.61 (0.25, 0.96)** | -0.02 (-0.15,0.11) | |  |
|  |  | **p < 0.001** | **p = 0.002** | p = 0.031 | **p = 0.001** | p = 0.75 | |  |
|  | **GSH/GSSG** | 0.28 (0.11, 0.68) | 0.56 (-0.06, 1.17) | 0.27 (0.00,0.54) | 0.20 (-0.22, 0.62) | -0.09 (-0.22,0.04) | |  |
|  |  | p = 0.16 | p = 0.076 | p =0.047 | p = 0.35 | p = 0.17 | |  |

^a^ Estimates, confidence intervals and p-values were obtained from regression models where log-transformed body fat compartment was the dependent variable and log-transformed thiol the main independent variable, with adjustment for age and lean mass. Estimates indicate % change in body fat compartment per % change in the thiol. Associations that are statistically significant after adjustment for multiple testing (see Methods for details) are in bold font.
