# Supplementary material for: The association of fasting plasma thiol fractions with body fat compartments, biomarker profile, and adipose tissue gene expression
Source: Amino Acids. 2022 Dec 21;55(3):313–23. doi: 10.1007/s00726-022-03229-2 (PMC10038976; doi:10.1007/s00726-022-03229-2)
Supplement: Supplementary file 4 — (DOCX 14 KB) [file 726_2022_3229_MOESM4_ESM.docx]

**Online Resource 5: Associations for fat compartments and select tGSH fractions adjusted for age, lean mass and plasma tCys^a^**

| **tGSH fraction** | **Fat compartment** | **Beta (95 % CI)** | **p** |
| --- | --- | --- | --- |
| Protein-bound GSH | Total fat mass | 0.13 (-0.02, 0.27) | 0.081 |
|  | Android fat mass | 0.25 (0.02, 0.47) | 0.032 |
|  | Gynoid fat mass | 0.08 (-0.08, 0.24) | 0.29 |
|  | Android/Gynoid FM | 0.16 (0.02, 0.31) | 0.031 |
| Reduced GSH | Total fat mass | 0.39 (0, 0.78) | 0.052 |
|  | Android fat mass | 0.49 (-0.14, 1.13) | 0.12 |
|  | Gynoid fat mass | 0.37 (-0.05, 0.79) | 0.084 |
|  | Android/Gynoid FM | 0.12 (-0.31, 0.55) | 0.57 |

^a^ Estimates, confidence intervals and p-values were obtained from regression models where log-transformed body fat compartment was the dependent variable and log-transformed thiol the main independent variable, with adjustment for age, lean mass and plasma tCys. Estimates indicate % change in body fat compartment per % change in the thiol.
