# Supplementary material for: The association of fasting plasma thiol fractions with body fat compartments, biomarker profile, and adipose tissue gene expression
Source: Amino Acids. 2022 Dec 21;55(3):313–23. doi: 10.1007/s00726-022-03229-2 (PMC10038976; doi:10.1007/s00726-022-03229-2)
Supplement: Supplementary file 5 — (DOCX 19 KB) [file 726_2022_3229_MOESM5_ESM.docx]

**Online Resource 5: Regression estimates and confidence intervals for the association of selected thiols and fractions with plasma biomarkers^a^**

|  |  | **Glucose** | **Insulin** | **C-peptide** | **HOMA-IR** | **Total cholesterol** | **Apolipoprotein B** | **Apolipoprotein A1** | **Triglycerides** |
| --- | --- | --- | --- | --- | --- | --- | --- | --- | --- |
| **Cysteine** | **Total** | 0.11 (-0.18,0.40) | 0.39 (-0.73,1.52) | 0.15 (-0.61,0.9) | 0.51 (-0.69,1.7) | 0.21 (-0.16,0.58) | **0.64 (0.17,1.12)** | -0.48 (-0.95,-0.01) | 0.43 (-0.5,1.37) |
|  |  | p = 0.45 | p = 0.48 | p = 0.69 | p = 0.40 | p = 0.26 | **p = 0.009** | p = 0.045 | p = 0.35 |
|  | **Protein-bound** | 0.01 (-0.19,0.22) | 0.53 (-0.25,1.32) | 0.23 (-0.28,0.74) | 0.54 (-0.29,1.37) | 0.11 (-0.16,0.37) | 0.28 (-0.08,0.64) | -0.11 (-0.46,0.23) | 0.1 (-0.58,0.77) |
|  |  | p = 0.90 | p = 0.18 | p = 0.36 | p = 0.19 | p = 0.42 | p = 0.12 | p = 0.50 | p = 0.77 |
|  | **Free** | 0.17 (-0.06,0.39) | -0.06 (-0.98,0.87) | 0.02 (-0.58,0.61) | 0.11 (-0.87,1.09) | 0.21 (-0.09,0.51) | 0.51 (0.12,0.89) | -0.34 (-0.72,0.03) | 0.49 (-0.27,1.24) |
|  |  | p = 0.14 | p = 0.90 | p = 0.95 | p = 0.82 | p = 0.16 | p = 0.012 | p = 0.07 | p = 0.20 |
|  | **Disulfide (cystine)** | 0.18 (-0.08,0.44) | 0.07 (-0.98,1.12) | 0.13 (-0.54,0.8) | 0.25 (-0.85,1.36) | 0.02 (-0.32,0.37) | 0.55 (0.11,0.99) | -**0.57 (-0.96,-0.17)** | 0.39 (-0.48,1.25) |
|  |  | p = 0.16 | p = 0.89 | p = 0.70 | p = 0.64 | p = 0.89 | p = 0.016 | **p = 0.007** | p = 0.370 |
|  | **Reduced** | 0.12 (0.01,0.23) | 0.15 (-0.31,0.62) | 0.16 (-0.13,0.45) | 0.28 (-0.2,0.76) | 0.03 (-0.12,0.18) | **0.26 (0.07,0.45)** | -0.2 (-0.39,-0.02) | 0.36 (-0.01,0.72) |
|  |  | p = 0.027 | p = 0.50 | p = 0.28 | p = 0.25 | p = 0.69 | **p = 0.010** | p = 0.034 | p = 0.058 |
|  | **Reduced cys/cystine** | 0.15 (0.01,0.3) | 0.25 (-0.37,0.86) | 0.23 (-0.15,0.62) | 0.4 (-0.24,1.04) | 0.04 (-0.16,0.25) | 0.26 (0,0.53) | -0.16 (-0.41,0.1) | 0.49 (0,0.97) |
|  |  | p = 0.039 | p = 0.420 | p = 0.226 | p = 0.210 | p = 0.657 | p = 0.054 | p = 0.225 | p = 0.049 |
| **Glutathione** | | | | | |  |  |  |  |
|  | **Protein-bound** | 0.01 (-0.03,0.05) | 0.17 (0.02,0.32) | 0.1 (0.01,0.2) | 0.18 (0.02,0.33) | 0.01 (-0.04,0.06) | 0.05 (-0.02,0.12) | -0.03 (-0.09,0.04) | 0.1 (-0.03,0.23) |
|  |  | p = 0.585 | p = 0.028 | p = 0.038 | p = 0.027 | p = 0.713 | p = 0.125 | p = 0.439 | p = 0.123 |
|  | **Reduced** | 0.07 (-0.04,0.18) | 0.13 (-0.31,0.57) | 0.12 (-0.16,0.4) | 0.2 (-0.27,0.66) | 0.12 (-0.02,0.26) | **0.25 (0.06,0.43)** | -0.11 (-0.29,0.08) | 0.33 (-0.02,0.68) |
|  |  | p = 0.225 | p = 0.554 | p = 0.394 | p = 0.394 | p = 0.080 | **p = 0.010** | p = 0.241 | p = 0.066 |

^a^ Estimates, confidence intervals and p-values were obtained from regression models where log-transformed biomarker was the dependent variable and log-transformed thiol the main independent variable, with adjustment for age. Estimates indicate % change in body fat compartment per % change in the thiol. Associations that are statistically significant after adjustment for multiple testing (see Methods for details) are in bold font.
