# Supplementary material for: The association of fasting plasma thiol fractions with body fat compartments, biomarker profile, and adipose tissue gene expression
Source: Amino Acids. 2022 Dec 21;55(3):313–23. doi: 10.1007/s00726-022-03229-2 (PMC10038976; doi:10.1007/s00726-022-03229-2)
Supplement: Supplementary file 6 — (DOCX 16 KB) [file 726_2022_3229_MOESM6_ESM.docx]

**Online Resource 6: Correlation coefficients and p-values for cysteine fractions and adipose tissue gene expression^a^**

|  | **Total cysteine** | | **Free cysteine** | | **Reduced cysteine** | | **Cystine** | | **Protein-bound GSH** | | **Reduced GSH** | |
| --- | --- | --- | --- | --- | --- | --- | --- | --- | --- | --- | --- | --- |
| **Adipose tissue mRNA** | r | p | r | p | r | p | r | p | r | p | r | p |
| *CPT1A* | 0.20 | 0.42 | -0.03 | 0.91 | 0.40 | 0.091 | **0.68** | **0.001** | -0.05 | 0.85 | 0.01 | 0.99 |
| *SREBP* | -0.15 | 0.55 | -0.01 | 0.97 | 0.17 | 0.49 | 0.01 | 0.95 | 0.01 | 0.59 | -0.13 | 0.61 |
| *SCD1* | -0.16 | 0.50 | -0.22 | 0.37 | 0.28 | 0.25 | 0.01 | 0.97 | 0.15 | 0.54 | -0.33 | 0.17 |
| *ACACA* | -0.28 | 0.25 | 0.03 | 0.91 | 0.25 | 0.30 | -0.08 | 0.76 | 0.18 | 0.45 | -0.39 | 0.10 |
| *DGAT1* | -0.02 | 0.93 | 0.07 | 0.78 | -0.11 | 0.65 | -0.26 | 0.29 | 0.10 | 0.70 | 0.20 | 0.42 |
| *FASN* | -0.35 | 0.15 | -0.01 | 0.97 | 0.01 | 0.97 | -0.29 | 0.23 | 0.08 | 0.75 | -0.15 | 0.53 |
| *PPARG* | 0.02 | 0.93 | -0.29 | 0.22 | -0.53 | 0.021 | -0.40 | 0.086 | 0.16 | 0.50 | -0.19 | 0.44 |
| *LEP* | 0.45 | 0.053 | -0.30 | 0.21 | -0.14 | 0.57 | 0.17 | 0.49 | 0.51 | 0.03 | -0.13 | 0.60 |

^a^ Spearman’s correlation coefficients for plasma total cysteine fractions and select mRNA transcripts in adipose tissue.
